# A Synthetic Riboswitch to Regulate Haloarchaeal Gene Expression

**DOI:** 10.3389/fmicb.2021.696181

**Published:** 2021-06-15

**Authors:** Johannes Born, Kerstin Weitzel, Beatrix Suess, Felicitas Pfeifer

**Affiliations:** ^1^Microbiology and Archaea, Darmstadt, Germany; ^2^Synthetic RNA Biology, Department of Biology, Technical University Darmstadt, Darmstadt, Germany; ^3^Centre of Synthetic Biology, Technical University Darmstadt, Darmstadt, Germany

**Keywords:** theophylline-dependent riboswitch E, *Haloferax volcanii*, transcriptional regulator GvpE, haloarchaeal Shine-Dalgarno sequence, gas vesicle gene promoter

## Abstract

In recent years, synthetic riboswitches have become increasingly important to construct genetic circuits in all three domains of life. In bacteria, synthetic translational riboswitches are often employed that modulate gene expression by masking the Shine-Dalgarno (SD) sequence in the absence or presence of a cognate ligand. For (halo-)archaeal translation, a SD sequence is not strictly required. The application of synthetic riboswitches in haloarchaea is therefore limited so far, also because of the molar intracellular salt concentrations found in these microbes. In this study, we applied synthetic theophylline-dependent translational riboswitches in the archaeon *Haloferax volcanii*. The riboswitch variants A through E and E^∗^ were chosen since they not only mask the SD sequence but also the AUG start codon by forming a secondary structure in the absence of the ligand theophylline. Upon addition of the ligand, the ribosomal binding site and start codon become accessible for translation initiation. Riboswitch E mediated a dose-dependent, up to threefold activation of the *bgaH* reporter gene expression. Raising the salt concentration of the culture media from 3 to 4 M NaCl resulted in a 12-fold increase in the switching capacity of riboswitch E, and switching activity increased up to 26-fold when the cultivating temperature was reduced from 45 to 30°C. To construct a genetic circuit, riboswitch E was applied to regulate the synthesis of the transcriptional activator GvpE allowing a dose-dependent activation of the *mgfp6* reporter gene under *P*_*pA*_ promoter control.

## Introduction

Inducible expression systems and especially synthetic genetic circuits allow to regulate gene expression by external molecules. A precise control of the protein biosynthesis allows to study the function of proteins at a defined state of growth. The addition of a specific inducer enables to switch on or off the expression of the gene of interest in a dose-dependent manner, and an adjustment of the expression level and thus the amount of protein produced is possible. In haloarchaea, adapted to molar concentrations of NaCl, the inducible tryptophan promoter *p.tna* and the inducible K^+^-dependent promoter *P*_*kdp*_ are available for an external conditional control of transcription (Large et al., 2007; Allers et al., 2010; Kixmuller and Greie, 2012). However, the control of translation by synthetic riboswitch elements has not yet been implemented in haloarchaea.

Riboswitches are *cis*-regulatory RNA structural elements consisting of an aptamer domain (sensor domain) as well as an expression platform (regulator domain) (Breaker, 2012; Mellin and Cossart, 2015). The complex three-dimensional structure of the aptamer domains enables the binding of their respective ligands. Interaction with the ligand leads to a conformational change of the aptamer that is directly transmitted to the expression platform controlling the expression of the following gene(s). The ligand binding domain is evolutionary conserved in secondary structure and in the nucleotides involved in ligand binding, whereas the expression platforms are less conserved. In bacteria, natural riboswitches influence transcription or translation. Riboswitches acting at the transcription level form a Rho-independent transcription terminator (e.g., guanine riboswitch *xpt*) or resolve it (adenine-activated riboswitch *ydhL* in *Bacillus subtilis*) after binding of the ligand (Mandal and Breaker, 2004). Bacterial translational riboswitches control the expression predominantly by masking the ribosomal binding site or Shine-Dalgarno (SD) sequence in the 5′ untranslated region (5′UTR) (Breaker, 2012).

Bacteria contain a large number of different natural riboswitches that are divided into nearly 40 classes (McCown et al., 2017). The riboswitches are able to bind amino acids, enzymatic cofactors, nucleic bases or inorganic ligands (Lunse et al., 2014). Riboswitches with affinity for adenosyl cobalamin, thiamine pyrophosphate (TPP) and flavin mononucleotide (FMN) have been identified (Mironov et al., 2002; Nahvi et al., 2002; Winkler et al., 2002). In eukaryotes, TPP riboswitches are involved in the splicing process (Wachter, 2010). Natural riboswitches regulating transcription, translation or splicing often serve as templates for the construction of synthetic ones (Topp and Gallivan, 2008a,b; Topp et al., 2011). In both eukaryotes and bacteria, synthetic riboswitches are easy-to implement, robust-operating regulatory elements to control translation (Suess et al., 2003; Breaker, 2012; Rudolph et al., 2013; Mellin and Cossart, 2015). The successful regulation of eukaryotic translation has been achieved by, e.g., inserting an *in vitro* selected aptamer between the cap structure and the AUG start codon (Suess et al., 2003).

In archaea, the only natural riboswitch experimentally determined is the fluoride-responsive riboswitch of the hyperthermophilic archaeon *Thermococcus kodakarensis*, presumably regulating the translation of genes involved in fluoride export and thus detoxification (Speed et al., 2018). Non-coding RNAs including kink-turn RNA motifs similar to bacterial riboswitches have been observed in *Pyrococcus abyssi* (Phok et al., 2011). Moreover, putative riboswitches with similarities to the eukaryotic or bacterial representatives have been determined in bioinformatic studies of archaeal genomes (Weinberg et al., 2010; Gupta and Swati, 2019). For example, a cyclic di-AMP-like riboswitch was predicted in the extremely halophilic archaeon *Halobacterium salinarum* (*Hbt. salinarum*). To date, synthetic riboswitches are rarely used to control translation in archaea. In the methanogen *Methanosarcina acetivorans* a ligand-specific and dose-dependent repression of gene expression has been achieved using the tetracycline-dependent riboswitch (Demolli et al., 2014). The stem-length, stability and the partial inclusion of the ribosomal binding site into the stem of the riboswitch determines the repression of the β-lactamase gene expression (Demolli et al., 2014). However, in the haloarchaeon *Haloferax volcanii*, fusion of the tetracycline riboswitch to the 5′ end of the leaderless dihydrofolate reductase transcript resulted in a complete repression of translation both in the presence or absence of tetracycline (Hering et al., 2009). The latter result suggests a high stability of this riboswitch at salt concentration of of 2.9 M KCl in *Hfx. volcanii*.

Haloarchaea such as *Hfx. volcanii* and *Hbt. salinarum* thrive in habitats containing 2–5.5 M NaCl. To adapt to these hypersaline conditions, haloarchaea use the salt-in strategy. The uptake of potassium and chloride ions protects the cells from dehydration in the hypersaline environment (Dennis and Shimmin, 1997; Oren, 1999). *Hbt. salinarum* forms gas vesicles for passive flotation in the aquatic milieu. The 14 *gvp* genes involved in *Hbt. salinarum* PHH1 are found in the p-vac region (plasmid-borne gas vacuole coding region) and arranged in two oppositely oriented gene clusters p-*gvpACNO* and p-*gvpDEFGHIJKLM* that are expressed throughout growth (Supplementary Figure 1; Englert et al., 1992a,b). A second *gvp* gene cluster, c-vac, leads to gas vesicle formation in the stationary growth only (Englert et al., 1992a). Both vac regions are related and harbor similar *gvp* genes in an identical arrangement. The transcription of p-vac is driven by the four promotors *P*_*pA*_, *P*_*pO*_, *P_*pD*_*, and *P*_*pF*_ (Hofacker et al., 2004) with growth-phase depended activities as determined using *mgfp6* encoding a green fluorescent protein as reporter gene (Born and Pfeifer, 2019). The *P*_*pA*_ promotor, responsible for the expression of p-*gvpACNO* encoding the major gas vesicle structural proteins GvpA and GvpC, shows the highest activity followed by *P*_*pO*_ (Hofacker et al., 2004; Born and Pfeifer, 2019). The earlier promoter analyses were done in *Hfx. volcanii* transformants using *bgaH* encoding a halophilic β-galactosidase as reporter gene (Holmes et al., 1997; Gregor and Pfeifer, 2001). The two promoters *P*_*pD*_ and *P*_*pF*_ exhibit lower activities (Born and Pfeifer, 2019). *P*_*pF*_ drives the transcription of p-*gvpFGHIJKLM* (Offner et al., 2000), whereas *P*_*pD*_ is responsible for the transcription of *p-gvpDE* encoding the two regulatory proteins GvpD and GvpE (Englert et al., 1992a,b; Hofacker et al., 2004). Both *P*_*pA*_ and *P*_*pD*_ are stimulated by the transcriptional activator GvpE resulting in 10- or 8-fold enhanced activities when the strong cGvpE activator derived from c-vac is used for these studies (Born and Pfeifer, 2019). The GvpE-mediated activation requires a 20 nt upstream activating sequence (UAS) located upstream of the TATA-box and BRE, the transcription factor B recognition element (Bauer et al., 2008; Marschaus and Pfeifer, 2012). The presence of GvpD reduces the amount of GvpE and thus the amount of gas vesicles (Englert et al., 1992b; Zimmermann and Pfeifer, 2003; Schmidt and Pfeifer, 2013). Except for p-*gvpO* that is transcribed as leaderless transcript, all other p-*gvp* transcripts contain a 5′UTR. A deletion of 5′UTR_*A*_, 5′UTR_*D*_, or 5′UTR_*F*_ results in a 2.4–4.5-fold increase of translation, suggesting that the 5′UTRs contribute to the regulation of the expression (Born and Pfeifer, 2019). The p-*gvpA* gene lacks a SD sequence in the 20-nt 5′UTR_*A*_, whereas all other p-*gvp* genes contain a SD sequence upstream of the respective reading frame (Sartorius-Neef and Pfeifer, 2004). The haloarchaeal consensus SD sequence is 5′-GGAGGUGA-3′, and the experimental analyses imply that the translation efficiency is influenced by the complementarity to the anti-SD in the 16S rRNA, but also by the distance of the SD sequence to the AUG start codon (Sartorius-Neef and Pfeifer, 2004). However, in other cases the putative haloarchaeal SD sequence had no influence on translation and question the importance of SD sequences for haloarchaea (Brenneis et al., 2007; Brenneis and Soppa, 2009).

The leaderless p-*gvpO* transcript is efficiently translated. Leaderless transcripts are not rare in haloarchaea; in *Hbt. salinarum*, 30% of mRNAs are leaderless, and in the moderately haloarchaeon *Hfx. volcanii* even 2/3rd of the transcripts (Slupska et al., 2001; Torarinsson et al., 2005; Brenneis and Soppa, 2009). One of the most prominent leaderless transcripts is the ferredoxin (*fdx)* mRNA starting at a guanosine directly upstream of the AUG start codon (Pfeifer et al., 1993). The translation of leaderless transcripts is more efficient compared to the leader-containing transcripts (Sartorius-Neef and Pfeifer, 2004; Born and Pfeifer, 2019). Presumably, translation occurs by a distinct mechanism as identified in bacteria, where the initiation of translation of leaderless transcripts involves an undissociated 70S ribosome and requires the recruitment of the initiator tRNA by IF2 and IF3 for complex formation with the mRNA (Moll et al., 2002, 2004; Andreev et al., 2006). Overall, haloarchaeal gene expression is regulated at the level of transcription initiation by regulator proteins, but also by sequences of the 5′UTRs that influence the translation.

**FIGURE 1:**
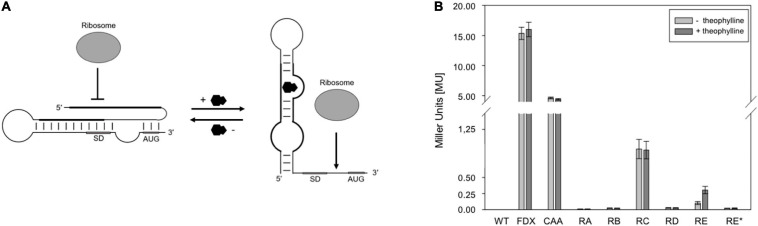
Test of theophylline-dependent riboswitches A through E and E* in *Hfx. volcanii*. **(A)** Proposed mechanism of a theophylline-dependent riboswitch. In the absence of theophylline (black molecule), the SD sequence and AUG start codon are buried in a secondary structure. Upon binding of theophylline to the aptamer domain, the SD sequence and the AUG start codon are accessible to the ribosome. SD, SD sequence; AUG, AUG start codon (modified after Rudolph et al., 2015). **(B)** Experimental data on the switching behavior of theophylline-dependent riboswitches A through E, and E* in *Hfx. volcanii*. The *bgaH* reporter gene was placed under the control of one of the six theophylline-dependent riboswitches and the BgaH activity was determined by ONPG assay in the respective transformants after 36 h of growth (37°C; OD_600_ of 1.2) in the presence or absence of 2 mM theophylline. The BgaH activity is given in Miller units (Supplementary Table 3). WT, *Hfx. volcanii* WR340 wild type; FDX, leaderless *bgaH* expression in pFDXJB20 transformants; CAA, transformants containing pFDXJB20_CAA and therefore CAA repeats instead of theophylline aptamer; RA, transformants containing riboswitch A; RB, riboswitch B; RC, riboswitch C; RD, riboswitch D; RE, riboswitch E; RE*, riboswitch E* inserted in plasmid pFDXJB20_R. The experiments were performed in triplicates on three different days.

To achieve an efficient external control of the haloarchaeal translation, the application of synthetic riboswitches might be useful. Good candidates are the synthetic theophylline-dependent riboswitch variants A through E and E^∗^ designed for different bacteria in the group of J. P. Gallivan. These variants contain engineered expression platforms to control ribosome binding and mainly differ in the nucleotide sequence of the bacterial SD sequence contained in these elements and its distance from the AUG start codon. In addition to *Escherichia coli* (Topp and Gallivan, 2008b), these theophylline-dependent riboswitches are used for example in *Synechococcus elongatus* (Nakahira et al., 2013), *Streptomyces coelicolor* (Rudolph et al., 2013), *Mycobacterium tuberculosis* (Seeliger et al., 2012), and *Francisella novicida* (Reynoso et al., 2012). The ability to regulate translation is based on the prediction that these riboswitches mask the AUG start codon and the SD sequence by forming a stable RNA structure in the absence of theophylline (Figure 1A; Lynch et al., 2007; Topp and Gallivan, 2008a; Topp et al., 2011). Binding of theophylline to the aptamer domain leads to the structural rearrangement, allowing the ribosome to interact with the single-stranded translation initiation region (Figure 1A; Lynch et al., 2007; Topp and Gallivan, 2008a; Topp et al., 2011).

In this report, we investigated the synthetic theophylline-dependent riboswitch variants in *Hfx. volcanii* to determine their application in an inducible expression system. *Hfx. volcanii* is moderately halophilic, easy to transform and grows faster than *Hbt. salinarum* (generation time: 4 h vs. 7 h). One of the six riboswitch variants tested influenced the expression of the *bgaH* reporter gene in response to theophylline. This riboswitch E was further characterized in respect to the kinetics, the dependence on salt concentration and the cultivation temperature. In addition, riboswitch E was used in a genetic circuit where the expression of *gvpE* was induced in response to the theophylline concentration applied. The different amounts of GvpE in turn activated the *P*_*pA*_ promoter driving the expression of the *mgfp6* reporter gene resulting in fluorescent cells. Indeed, the fluorescence increased in response to the rising concentrations of theophylline.

## Materials and Methods

### Strains and Cultivation Conditions

*E. coli* Top10F’ (Invitrogen, Carlsbad, United States) was used for all plasmid manipulations. Transformants of this strain were cultivated at 37°C overnight in Luria-Bertani broth supplemented with 100 μg/ml ampicillin. *Hfx. volcanii* WR340 (Bitan-Banin et al., 2003) and the respective transformants were grown at 42 or 37°C in media containing 3 M NaCl, 150 mM MgSO_4_, 50 mM KCl, 10 nM MnCl_2_, 25 mM Tris/HCl pH 7.2, 0.5% (w/v) tryptone, 0.3% (w/v) yeast extract, and 0.02% (w/v) histidine. To test the effect of the different salt concentrations on the activity of the theophylline-dependent riboswitches, media with salt concentrations of 1.5, 2.0, 3.0, 3.5, or 4.0 M NaCl were used. For riboswitch activation, theophylline was added to the salt media in concentrations of up to 4 mM. *Hfx. volcanii* transformants were selected on solid media containing 6 mg/ml lovastatin (Lam and Doolittle, 1989). Cultures plated on solid media containing 1.8% (w/v) agar were incubated in plastic bags at 42°C under humid conditions for 4–5 days.

### Construction of Plasmids and Transformation of *Hfx. volcanii*

**FIGURE 2:**
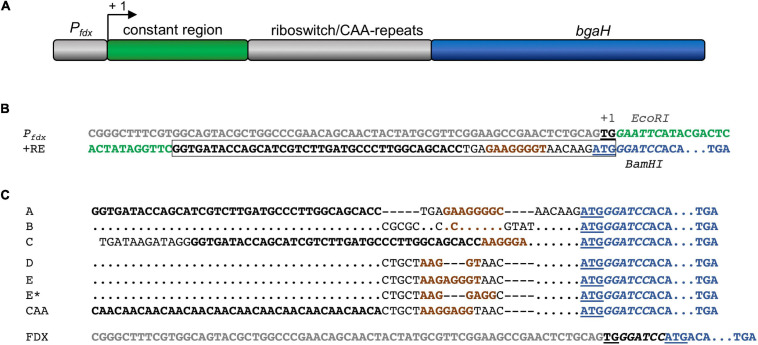
*P*_*fdx*_ promoter and synthetic 5′UTR to study theophylline-dependent *bgaH* translation in *Hfx. volcanii*. **(A)** Schematic representation of the *P_*fdx*_-*riboswitch-*bgaH* fragments. A constant region and the respective riboswitch (or CAA repeat) were inserted between the *P*_*fdx*_ promoter and the *bgaH* reading frame. The transcription start site is marked +1. **(B)** DNA sequences of *P*_*fdx*_ and riboswitch E. The sequence of *P*_*fdx*_ is given in gray and the constant region of the 5′UTR is marked in green. The transcriptional start (TG) is bold and underlined and the ATG start of the *bgaH* reading frame in blue and underlined. The theophylline aptamer is shown in bold, and the bacterial SD sequence in riboswitch E is highlighted in brown. The complete riboswitch E is boxed. The recognition sequences of *Eco*RI and *Bam*HI surrounding the constant region and the riboswitch are in *italics*. The open reading frame of *bgaH* is highlighted in blue. **(C)** DNA sequence of the fusion of the respective riboswitch sequences to the open reading frame of *bgaH*. The aptamer domain (or CAA-repeats) are highlighted in bold and the bacterial SD sequence is shown in brown. The ATG start is underlined and the *Bam*HI-site in *italics*, and the open reading frame of *bgaH* is highlighted in blue. Dots mark the same nucleotides as shown in the riboswitch A sequence. Dashes indicate gaps in the sequence. For the location of the haloarchaeal SD sequences in these sequences compare Table 1. In the case of plasmid pFDXJB20 (FDX), the *P*_*fdx*_ promoter sequence is highlighted in gray, while the ATG start is underlined and the *BamHI* is shown in *italics*.

Different plasmids were constructed to investigate the regulation of haloarchaeal translation by the six theophylline-dependent riboswitches. As intermediate plasmid served pLacZJB20. Plasmid pLacZJB20 was constructed following the model of pP_*fdx*_JB18 (Born and Pfeifer, 2019) and was generated by NEBuilder^®^ HiFi DNA Assembly Master Mix (New England Biolabs). In contrast to pP_*fdx*_JB18, pLacZJB20 carries the *bgaH* reading frame (encoding a halophilic β-galactosidase) instead of *smRS-gfp* as reporter, and *lacZ* instead of the *P*_*fdx*_ promoter (Supplementary Table 1). The restriction sites *Eco*RI and *Bam*HI present in the original *bgaH* reading frame were removed by silent mutations. To test the six riboswitches in *Hfx. volcanii, lacZ* of pLacZJB20 was replaced *via Nco*I and *Bam*HI by the *P*_*fdx*_ promoter resulting in pFDXJB20_Eco (Supplementary Table 1). The P*_*fdx*_* promoter fragment contains the transcriptional start of the ferredoxin gene (*fdx*) and an *Eco*RI restriction site, so that *P*_*fdx*_ is surrounded by *Nco*I and *Eco*RI sites, and the *bgaH* reading frame by *Bam*HI and *Kpn*I sites (see Supplementary Figure 2A). Subsequently the ATG start of *bgaH* was deleted by mutagenesis PCR resulting in plasmid pFDXJB20_Eco_ΔATG. The *Eco*RI and *Bam*HI sites were used to insert the riboswitch elements. The respective *Eco*RI-*Bam*HI fragments contained a constant region of 28 bp and the sequence of one of the six riboswitches including the ATG start. The fragment was inserted between *P*_*fdx*_ and *bgaH* to yield pFDXJB20_R (R represents the sequence of one of the six riboswitches) (Supplementary Figure 2A). As control, plasmid pFDXJB20_CAA was generated containing the CAA fragment inserted in pFDXJB20_Eco_ΔATG in the same way (Supplementary Table 1). The CAA fragment consists of a non-structured CAA repeat sequence (Hanson et al., 2003) instead of a theophylline-dependent aptamer between the constant region and the expression platform of the theophylline-dependent riboswitch E (Figure 2). The resulting plasmids harbor *bgaH* under control of *P*_*fdx*_ and one of the six theophylline-dependent riboswitches or the CAA-repeat sequence upstream and adjacent to *bgaH*. The sequences of *P*_*fdx*_ and of the six theophylline-dependent riboswitches (including the constant region) as well as of the CAA fragment are shown in Figures 2B,C. The respective *Eco*RI-*Bam*HI fragments were obtained by annealing oligonucleotides containing the desired DNA region as sense or antisense strand plus nucleotide overhangs for the restriction sites *Eco*RI and *Bam*HI (Supplementary Table 2). For hybridization, 100 μM per oligonucleotide were mixed in a 1:1 ratio, incubated for 5 min at 95°C and slowly cooled down at room temperature. The resulting DNA fragments were then phosphorylated and inserted into the plasmids. Plasmid pFDXJB20 (Supplementary Figure 2B) contains a *Bam*HI site directly upstream of the ATG start of *bgaH*, separating the transcription start site and the ATG only by this *Bam*HI site (Figure 2C). The plasmid served to investigate the translation of leaderless *bgaH* transcripts.

Plasmid pPAJB20 (Supplementary Figure 2C and Supplementary Table 1) was constructed to investigate the activation of *P_*pA*_ via* the riboswitch E-controlled synthesis of GvpE. The ATG start of *gvpE* was deleted in pPDPAJB18+E (Born and Pfeifer, 2019), and an *Eco*RI site was inserted between *gvpE* and the *Spe*I site. Both steps were performed by mutagenesis PCR. The oligonucleotides used are listed in Supplementary Table 2. Subsequently, riboswitch E was inserted *via* the *Eco*RI and *Spe*I sites (RE) resulting in pPAJB20.

All constructs were verified by DNA sequence determination (Eurofins Genomics Germany GmbH, Ebersberg). *Hfx. volcanii* WR340 was transformed as described by Pfeifer and Ghahraman (1993) and the plasmids of the resulted transformants controlled by PCR and DNA sequencing.

### Quantification of GFP Fluorescence and Western Blot Analysis

The mGFP6 fluorescence of *Hfx. volcanii* WR340 transformants containing plasmid pPAJB20 and Western blot analysis to detect the different amounts of GvpE *in vivo* were performed as described by Born and Pfeifer (2019).

### Determination of BgaH Activity by ONPG Test

The activity of the BgaH was measured by ONPG test. The assay is based on the β-glycosidic cleavage of the colorless ONPG (ortho-nitrophenyl-β-D-galactopyranoside) to galactose and o-nitrophenol (ONP), resulting in a yellow color. The intensity of the coloration depends on the amount of BgaH formed as well as the reaction time and can be measured at a wavelength of 420 nm. To determine the BgaH activity, 50 ml complex medium was inoculated with a preculture of the respective *Hfx. volcanii* transformant (OD_600_ of 0.6) to a starting OD_600_ of 0.02 and incubated at 180 rpm and the respecting incubation temperature until OD_600_ 1–1.2 was reached. The salinity of the media and the incubation temperature used for the cultivation varied in the different experiments. The optical density of each culture was determined and cells of a 1 ml suspension harvested by centrifugation at 5.000 rpm for 15 min. The supernatant was removed and the cells were placed in 300 μl digestion buffer (2.5 M sodium chloride, 10 μM manganese chloride, 50 mM Tris-HCl; pH 7.2, 0.1% TritonX-100 and 1 mg/ml DNase) and incubated at 37°C for 30 min. Each preparation was mixed with 700 μl ONPG test buffer (2.5 M sodium chloride, 10 μM manganese chloride, 50 mM Tris-HCl; pH 7.2, 0.1% beta-mercaptoethanol, 2 mg/ml ONPG). After incubation (between 30 min and 4 h at 37°C) the reaction was stopped by the addition of 100 μl 0.5 M EDTA and the absorbance determined at 420 and 550 nm. The Miller Units were calculated using the following formula:

$$1\,\mathrm{Miller}\,\mathrm{Unit}\,\frac{1000*\text{abs}\,420-\left(1.75*\text{abs}\,550\right)}{t*v*\mathrm{abs600}}$$

abs420: absorbance ONP, abs550: scattering of cell debris, t: reaction time [min], v = culture volume used [ml], abs600: optical density.

## Results

### Application of Theophylline Riboswitches to Regulate the Expression of *bgaH*

In conditional gene expression systems, the gene expression is regulated by the addition of a specific ligand. Depending on the bacterial species, the ligand used may affect the metabolism. To determine a putative influence of theophylline on the growth of *Hfx. volcanii*, different theophylline concentrations (0, 1, 2, 3, 4, 5, or 10 mM) in 3 M salt media (3 M NaCl) were used and the cultures grown at 42°C for 85 h. In almost all cases, the growth curves were similar to cells cultivated without theophylline (Supplementary Figure 3A). The slowest growth was observed with the culture containing 10 mM theophylline, but also this culture reached similar OD_600_ values after 80 h of growth in the stationary phase.

To study the effect of theophylline on the haloarchaeal gene expression, plasmid pFDXJB20 was used containing the *bgaH* reading frame expressed directly under *P*_*fdx*_ promoter control and resulting in a leaderless *bgaH* transcript (Figure 2C and Supplementary Table 1). *Hfx. volcanii* pFDXJB20 transformants were grown in 3 M salt media at 42°C to OD_600_ 1.2 in the absence or presence of 2 mM theophylline, and the *bgaH* expression was measured by ONPG assay in Miller Units [MU] (Supplementary Figure 3B). Both cultures produced similar amounts of BgaH (15 MU), suggesting that theophylline had no negative influence on growth or expression.

The function of the six theophylline-dependent riboswitch variants A through E and E^∗^ (Lynch et al., 2007; Topp and Gallivan, 2008a,b) was tested by inserting the respective sequences as *Eco*RI and *Bam*HI fragment upstream of the *bgaH* reading frame in pFDXJB20_Eco_ΔATG to yield the respective pFDXJB20_R plasmid (Supplementary Figure 2A). The original ATG start of *bgaH* is deleted in pFXDJB20_Eco_ΔATG, and the riboswitch sequence included a new ATG start. As a result, the riboswitch is placed between the transcriptional start and overlaps the novel start codon of the *bgaH* reading frame. The synthetic 5′UTR was composed of a 28-nt long constant region upstream of the riboswitch sequence that was identical in all these constructs (Figure 2). As positive control served plasmid pFDXJB20_CAA containing non-structured CAA repeats (Hanson et al., 2003) instead of the sequence of the aptamer (Figure 2C). Transformants harboring these constructs were designated RA through RE and RE^∗^ (or CAA) according to the riboswitch sequence used to regulate *bgaH* expression. These transformants were cultivated at 37°C for 36 h to OD 1.2 in the presence or absence of 2 mM theophylline. The temperature of 37°C was chosen, since these riboswitches have the highest activation ratio at temperatures between 28 and 37°C as found for several bacteria (Topp et al., 2011; Rudolph et al., 2013). A high BgaH activity was observed with the CAA transformants producing transcripts with the non-structured CAA repeats in the 5′UTR (Figure 1B). Compared to the expression of the leaderless *bgaH* transcript produced in the FDX transformants (containing pFDXJB20) the activity was approximately 70% reduced (4 MU vs. 15 MU), but much larger compared to the activities found in the different riboswitch transformants. A very low BgaH activity (0.02 MU) was obtained with the RA, RB, RD, and RE^∗^ transformants, whereas RC transformants yielded with 0.95 MU a higher expression of *bgaH* (Figure 1B). The low expression observed in the RA, RB, RD, and RE^∗^ transformants suggested a stable riboswitch structure preventing the translation of *bgaH*. Differences in *bgaH* expression were not observed in the presence or absence of theophylline (Figure 1B) implying that theophylline was unable to destabilize the riboswitch structure to increase the translation. Riboswitch C appears to be less stable, but the presence of theophylline did not enhance the BgaH activity. In contrast, riboswitch E yielded a low expression of *bgaH* in the culture lacking theophylline, and a threefold higher expression in the presence of theophylline (Figure 1B) suggesting that riboswitch E changed the translational activity in response to the ligand.

To compare the regulatory effect of riboswitch E when different amounts of transcript are present, the strong *P*_*fdx*_ promoter was replaced by the rRNA promoter *P2*, the intermediate promoter *P*_*pA*_, or the weak promoter *P*_*pD*_ (Born and Pfeifer, 2019), and the expression of *bgaH* was analyzed. In all three cases, BgaH activity was not detectable in the presence of theophylline, implying a repression of the *bgaH* translation. It is likely that the activities of these promotors were too low to yield a detectable amount of BgaH in the presence of the synthetic 5′UTR including riboswitch E. Only the combination of *P*_*fdx*_ and riboswitch E resulted in a detectable amount of BgaH.

### Dose-Dependency and Switching Activity of Riboswitch E

**FIGURE 3:**
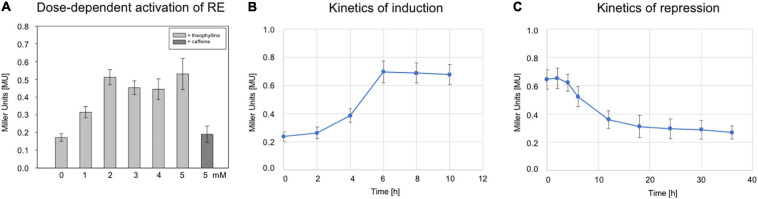
Characterization of riboswitch E. **(A)** Effect of different theophylline concentrations on *bgaH* expression controlled by riboswitch E. *Hfx. volcanii* RE transformants were grown in the presence of 0, 1, 2, 3, 4, or 5 mM theophylline to OD_600_ 1.2 (3 M salt media, 37°C), and the BgaH activity was determined in Miller units. As a control, 2 mM caffeine was used instead of theophylline. The experiments were performed in triplicates on three different days. **(B)** The *bgaH* expression and BgaH activity after the addition of theophylline. RE transformants were cultured for 8 h (3 M salt media, 37°C) and then theophylline was added to a final concentration of 5 mM. The BgaH activity was quantified after 2, 4, 6, 8, and 10 h of growth. **(C)** Repression of *bgaH* expression by removing theophylline. RE transformants were cultured for 8 h at 37°C in 3 M salt media containing 2 mM theophylline. Incubation of cells was continued for 36 h without theophylline and the BgaH activity was quantified after 2, 4, 6, 12, 18, 24, 30, and 36 h of growth. Shown are the mean values and standard deviations of two independent measurements with three biological replicates each.

To study the dose-dependent activation of riboswitch E, the RE transformants were grown to OD_600_ 1.2 at 37°C in 3 M salt media in the presence of different theophylline concentrations (0–5 mM). The *bgaH* expression increased up to 2 mM theophylline, and stayed almost constant at 0.5 MU with theophylline concentrations up to 5 mM (Figure 3A). Caffeine was applied as control ligand since its structure is very similar to theophylline but does not bind to the aptamer. Supplementation of the culture with caffeine did not activate the switch, demonstrating a ligand-specific activation. To determine the time required for the activation, the RE transformants were grown without theophylline for 8 h in 3 M salt media at 37°C. Then, theophylline was added to a final concentration of 2 mM and the BgaH activity determined over a period of 10 h (Figure 3B). A small increase in the BgaH activity already appeared after 4 h of theophylline supplementation, and the maximal BgaH activity was observed after 6 h of growth. To determine the kinetics of the repression, the RE transformants were grown in media containing 2 mM theophylline for 8 h, washed with salt water and resuspended in media without theophylline. Quantitation of the BgaH activity over 40 h of growth indicated that the activity decreased within 12 h and reached the level of the non-induced culture after 36 h (Figure 3C).

### Temperature and Salt-Dependency of Riboswitch E

**FIGURE 4:**
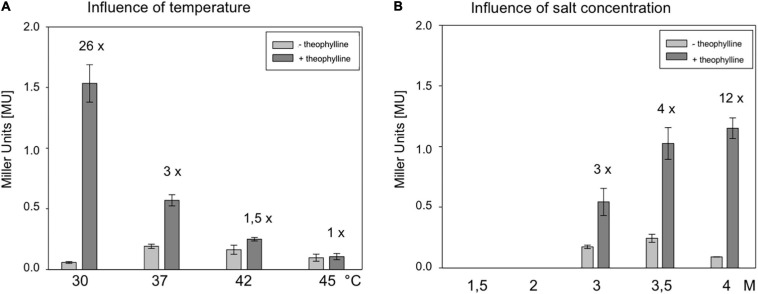
Characterization of the theophylline-dependent riboswitch E in respect to cultivation temperature and salt concentration of the medium. **(A)** Effect of temperature on the activation of riboswitch E in *Hfx. volcanii*. RE transformants were incubated for 48 h at 30, 37, 42, or 45°C in 3 M salt media in the presence or absence of 2 mM theophylline, and the BgaH activity was determined. **(B)** Effect of the salt concentration on the activation of riboswitch E in *Hfx. volcanii* transformants. RE transformants were cultured at 37°C for 37 h in media containing 1.5, 2, 3, 3.5, or 4 M NaCl in the presence or absence of 2 mM theophylline, and the BgaH activity was determined (Supplementary Table 4). All experiments were performed in triplicates on three different days.

To determine the influence of temperature, *Hfx. volcanii* WR340 was grown at 30, 37, 42, and 45°C in 3 M salt media (Supplementary Figure 4A). The optimal growth temperature appeared to be 45°C, followed by 42 and 37°C, whereas growth at 30°C was significantly slower. To determine an effect of temperature on BgaH activity, transformants containing pFDXJB20 (producing a leaderless *bgaH* transcript) were cultured at similar temperatures up to OD 1.2, and no significant effect was detected (Supplementary Figure 4B). The RE transformants (containing pFDXJB20_RE) were used to determine the effect of these temperatures on the switching activity of riboswitch E. The transformants were grown at these temperatures for 45 h in the presence or absence of 2 mM theophylline (Figure 4A). The strongest activation (26-fold) was observed at 30°C, and the higher the growth temperature, the lower was the activation of *bgaH via* riboswitch E (Figure 4A). At 45°C, riboswitch E was inactive. Thus, 30°C turned out to be the optimal temperature for a high switching activity of riboswitch E.

The influence of the intracellular salt concentration was tested by growing *Hfx. volcanii* WR340 in media containing 1.5, 2.0, 3.0, 3.5, or 4.0 M NaCl. Haloarchaea adapt to these salt concentrations by maintaining a similarly high salt KCl concentration in their cytoplasm. At 37°C, growth of *Hfx. volcanii* was optimal in 2.0 or 3.0 M NaCl containing media, whereas the growth is considerably reduced in media containing larger or smaller amounts of salt (Supplementary Figure 5A). Using pFDXJB20 transformants for these experiments, a strongly reduced BgaH activity was observed in cells grown at 1.5 and 2.0 M salt media (Supplementary Figure 5B), demonstrating the already described sensitivity of BgaH to salt concentrations lower than 3 M (Holmes et al., 1997). The RE transformants were grown at 37°C in these five different salt media in the presence or absence of 2 mM theophylline, and the BgaH activities were quantified after 37 h of growth (Figure 4B). Growth in 4 M salt medium activated the *bgaH* expression 12-fold, whereas the activation in 3 or 3.5 M salt media was only 3- or 4-fold. In contrast, RE transformants grown in 1.5 or 2 M salt media did not contain detectable BgaH activities (Figure 4B). Thus, the highest salt concentration (4 M) yielded the highest switching activity by theophylline. Taken together, growth at 30°C in 4 M salt media appeared to be the best conditions to support the switching activity of riboswitch E. The RE transformants were tested under these conditions in the presence or absence of theophylline. The cells were grown for 6 days to OD_600_ 1.2 and the BgaH activity was determined. The culture containing theophylline indicated a 13-fold activation compared to the activity in the non-induced state (4.0 ± 0.48 MU vs. 0.3 ± 0.01 MU). The BgaH activity determined was as high as the activity determined for the CAA transformant containing the non-structured aptamer in the 5′UTR of the *bgaH* transcript (4.4 MU).

### Construction of a Theophylline Dependent Expression System

**FIGURE 5:**
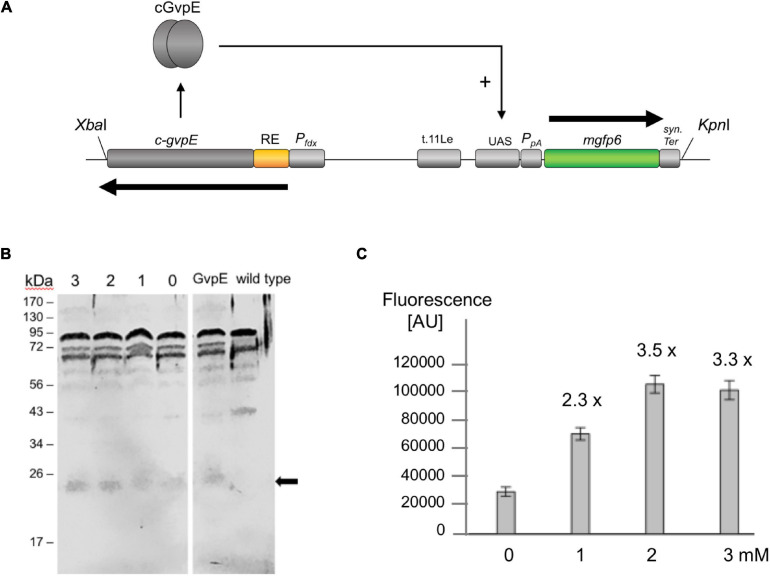
GvpE-mediated activation of *P*_*pA*_ under control of riboswitch E. **(A)** Genetic map of the *Xba*I-*Kpn*I fragment containing the relevant genes and regulatory structures of plasmid pPAJB20. The c-*gvpE* gene of the c-vac region of *Hbt. salinarum* is placed under control of *P*_*fdx*_ and riboswitch E (RE, yellow), and in the opposite orientation the reporter gene *mgfp6* is placed under control of *P*_*pA*_ including the UAS_*A*_ sequence required for GvpE-mediated activation of *P*_*pA*_. The *mgfp6* gene is surrounded by the two terminator sequences t.11Le and synTer to prevent read-through. Arrows depict the direction of transcription. **(B)** Western blot analysis to detect the amounts of GvpE in cells grown in the presence or absence of theophylline. Cells were grown to OD_600_ 1.2 and disrupted to isolate total proteins. Twenty μg of total protein each were separated by SDS-PAGE and transferred to a PVDF membrane. The detection of GvpE was done using a GvpE antiserum and a second antibody coupled with the fluorescent dye IRDye 800 CW (LI-COR). Western blots were inverted to black and white. The expected protein size of GvpE is indicated by an arrow. Numbers on the left are marker sizes in kDa. The numbers 1, 2, and 3 depict theophylline concentrations in mM; 0, without theophylline. GvpE, total protein of a GvpE-producing transformant, wild type, total proteins of *Hfx. volcanii* WR340. **(C)** *P*_*pA*_ activation in pPAJB20 transformants producing different amounts of GvpE. The pPAJB20 transformants were incubated with 0, 1, 2, or 3 mM theophylline in 3 M salt media at 37°C. The fluorescence of the cells was measured after 30 h of growth (OD_600_ of 0.6) and is given in arbitrary units (AU) (Supplementary Table 5). The experiments were performed in triplicates on three different days. 0, 1, 2, 3 mM designate the respective theophylline concentrations used.

The GvpE-inducible *P*_*pA*_ promoter of the p-vac region and the transcriptional activator cGvpE were used to assemble a genetic system under the control of theophylline-dependent riboswitch E. The *P*_*pA*_ activity was determined using the *mgfp6* reporter gene (Born and Pfeifer, 2019), and our aim was to control the amount of fluorescence by the external concentration of theophylline via controlling the synthesis of cGvpE by riboswitch E. Plasmid pPAJB20 contains c-*gvpE* expressed under the control of *P*_*fdx*_ and riboswitch E, and the *mgfp6* reporter gene controlled by the promoter *P*_*pA*_ including the UAS_*A*_ required for the activation by GvpE (Supplementary Figure 2C). mGFP6 is a GFP variant that functions well under the high intracellular salt condition of *Hfx. volcanii* (Born and Pfeifer, 2019). The c-*gvpE* and *mgfp6* genes are oppositely oriented and separated by the transcriptional terminator t.11Le (Supplementary Figure 2C and Figure 5A). Transformants carrying pPAJB20 should express c-*gvpE* depending on theophylline, and GvpE will activate *P*_*pA*_ driving the expression of the *mgfp6* reporter gene. To determine whether the amount of GvpE depends on the theophylline concentration, transformants containing pPAJB20 were grown in 3 M salt media supplied with different theophylline concentrations (0–3 mM). Total proteins were isolated and separated by SDS-polyacrylamide gel electrophoresis, and GvpE was determined by Western blot analysis using an antiserum raised against GvpE (Krüger et al., 1998). The amount of GvpE increased up to 2 mM theophylline, suggesting that the expression of *gvpE* was indeed regulated by theophylline (Figure 5B).

To quantify the *mgfp6* expression, the transformants were grown for 30 h up to OD 0.6 in 3 M salt media containing the different theophylline concentrations, and the fluorescence of the cells was determined. When grown without theophylline (0 mM), the PAJB20 transformants showed a twofold higher fluorescence (30,000 AU) compared to the basal activity of *P*_*pA*_ in PDPA transformants (18,000 AU) containing a similar plasmid but lacking *gvpE* (Born and Pfeifer, 2019) suggesting that the riboswitch did not completely prevent the expression of *gvpE*. The addition of 1, 2, or 3 mM theophylline resulted in a 2.3- to 3.5-fold increase of the GFP signal (Figure 5C), indicating that the *mgfp6* expression increased with the concentration of theophylline supplied. The maximal GvpE-mediated activation of *P*_*pA*_ (100,000 AU) reached half of the fluorescence determined for PDPA+E transformants (200,000 AU) producing a leaderless *gvpE* transcript controlled by *P*_*fdx*_ (Born and Pfeifer, 2019). The presence of a 5′UTR harboring riboswitch E and the constant region obviously reduced the translation of *gvpE* in PAJB20 transformants, as observed with other leader-containing transcripts.

## Discussion

The theophylline-dependent riboswitches A through E and E^∗^ were tested for their ability to regulate gene expression in the haloarchaeon *Hfx. volcanii*. Only riboswitch E was able to regulate the translation at the high intracellular salt concentrations present in haloarchaea. Riboswitch E was characterized in respect to the switching activity, the salt concentration of the media, and the cultivation temperature. In addition, a small genetic circuit was constructed to achieve a dependence of the *P*_*pA*_-dependent *mgfp6* reporter expression on the amount of the external ligand theophylline.

### Theophylline Activates Riboswitch E in *Hfx. volcanii*

Theophylline-dependent riboswitches have been used to regulate gene expression in a variety of bacterial species. Activation factors up to 260-fold are found after the addition of theophylline (Topp et al., 2011; Nakahira et al., 2013; Rudolph et al., 2013). In *Hfx. volcanii*, activation of the *bgaH* expression upon the addition of theophylline was only detected for riboswitch E (Figure 1B), whereas no activation by theophylline was observed for the riboswitch A through D, and E^∗^. Except for riboswitch C, all these riboswitches repressed the expression of *bgaH* in the absence and presence of theophylline, but a construct containing the non-structure-forming CAA repeats instead of the aptamer domain yielded a much higher expression (Figure 1B). These results imply that a stable RNA structure was formed by these riboswitches that impedes translation.

The riboswitches A through E and E^∗^ are optimized for use as inducible expression system in different bacteria. These variants mainly differ in the nucleotide sequence of the SD sequence and its distance to the AUG start codon (Table 1). Investigation of the Gram-positive bacterium *S. coelicolor* demonstrated that riboswitches with the highest number of nucleotides complementary to the anti-SD sequence near the 3′end of the 16S RNA yielded the strongest gene expression when the inducer was added (Rudolph et al., 2013, 2015). We assume that the nucleotide sequence and the distance of the putative haloarchaeal SD sequence from the AUG start codon also influenced the activation potential of these riboswitches in *Hfx. volcanii.* Such putative SD sequence were determined for the six theophylline-dependent riboswitches and match the haloarchaeal consensus sequence 5′-GGAGGUGA-3′ by 4–7 nt (Table 1). The distance of these putative haloarchaeal SD sequences to the AUG start codon varies from 2 to 7 nt. These sequences are not identical to the bacterial SD sequences in the riboswitches, but occur a few nucleotides further upstream or downstream (Table 1).

**TABLE 1:** Putative SD sequence in riboswitches A through E and E*.

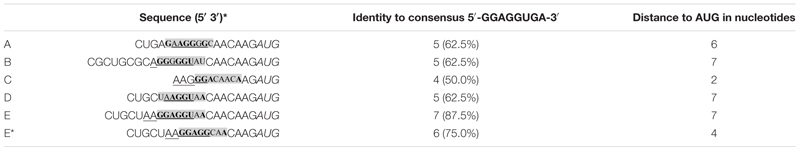

*Putative haloarchaeal SD sequences shaded in gray; the bacterial SD sequence is underlined. Nucleotides identical to the consensus sequence are in bold. The AUG start codon is in italics.

Previous investigations suggested that the translation efficiency in haloarchaea is influenced by the distance of the putative SD sequence from the AUG start codon and also by the complementarity to the anti-SD sequence in the 16S rRNA (Sartorius-Neef and Pfeifer, 2004). The core motif 5′-GGAGG-3′ at the 5′ end of the SD sequence has the major influence on the strength of the gene expression. Substitution of the p-*gvpH* SD sequence 5′-GGAGGUCA-3′ by 5′-UUAGGUCA-3′ results in a 90% reduction of the expression, although the remaining 5 nucleotides are not altered (Sartorius-Neef and Pfeifer, 2004). The haloarchaeal SD sequences in the riboswitch variants A, B, C, and D lack the core motif 5′-GGAGG-3′, but this motif is present in the riboswitch E and riboswitch E^∗^. In addition, riboswitch E contains with 5′-GGAGGUAA-3′ a nearly perfect SD sequence (SD_*E*_, 87.5% identity) in the optimal distance of 7 nt from the AUG start codon. Furthermore, SD_*E*_ is almost identical to the SD sequence of p-*gvpH* (SD_*H*_, 5′-GGAGGUCA-3′) that also occurs 7 nt upstream of the AUG codon, and mutations in SD_*H*_ result in a lower translation efficiency of p-*gvpH* (Sartorius-Neef and Pfeifer, 2004). The almost perfect SD_*E*_ in a distance of 7 nt to AUG could be one of the reasons that riboswitch E is active, whereas the other riboswitches are all inactive in haloarchaea.

In the case of riboswitch E^∗^, the putative haloarchaeal SD sequence (SD_*E*_^∗^) is with 75% identity less conserved (Table 1). Compared to riboswitch E, riboswitch E^∗^ yielded a much lower read-through translation and lacked a theophylline-induced activation of gene expression (Figure 1B). Both riboswitches are optimized for Gram-positive bacteria (Topp and Gallivan, 2008a,b; Topp et al., 2011). They derive from riboswitch D (that was not functional in haloarchaea) by inserting an additional AGG triplet in the case of riboswitch E, and replacing the triplet UAA by AGG in the case of riboswitch E^∗^ (Topp et al., 2011). As a result, E^∗^ and E contain the SD core region 5′-GGAGG-3′ required in the haloarchaeal SD-sequence for translation. However, the SD_*E*_ and SD_E__*_ sequences differ in their distance to the AUG start codon (7 nt in riboswitch E vs. 4 nt in riboswitch E^∗^), and this difference might be the reason for the much lower translation efficiency of riboswitch E^∗^. A minimal distance of 3 nt between the SD sequence and AUG start codon is required (Sartorius-Neef and Pfeifer, 2004). It is possible that the larger and optimal distance of SD_*E*_ allows the translation of the adjacent reading frame in haloarchaea. These results underline that the conservation and position of the SD sequence might be important for the translation of these genes.

Hypersaline conditions, i.e., the presence of large amounts of monovalent and multivalent ions, can affect RNA folding and RNA-ligand binding (Draper, 2004; Tan and Chen, 2011; Bizarro et al., 2012). Repulsion of the negative charges of RNA counteracts the folding of a compact structure. This folding is favored by shielding of the negative charges with positive ions (Draper, 2004). Since phosphate charges are more strongly shielded by counterions at high salt concentrations, the potential repulsion energy is lower, which stabilizes the RNA structures. These RNA structures can be very stable as shown for the tetracycline aptamer in *Hfx. volcanii* that was fused at the 5′-end of *dhfr* used as reporter gene (Hering et al., 2009). In the presence of tetracycline, it was expected to prevent translation initiation by forming a stable structure, whereas in the absence of tetracycline the riboswitch is open and allows translation as shown for *Saccharomyces cerevisiae* (Hanson et al., 2003). However, gene expression was not detectable under both conditions. Hering et al. (2009) assumed that due to the hypersaline environment, the aptamer structure was already stabilized in the absence of tetracycline. Similarly, the theophylline-dependent riboswitches examined here may be stabilized by the high intracellular salt concentration found in *Hfx. volcanii*. To what extent the intracellular salt influences the individual riboswitches and why only riboswitch E was activated should be investigated in further studies. It is conceivable that riboswitch E met the following three optimal conditions: (i) in the absence of theophylline ligand, a stable secondary structure was formed, (ii) this structure could be resolved by the addition of theophylline, (iii) after the conformational change induced by theophylline the putative SD sequence (with near-optimal identity to the consensus and optimal distance to the AUG) was single-stranded and accessible to the ribosome (Supplementary Figure 6).

### Cultivation Temperature and Salt Concentration Affect Riboswitch E

The moderately halophilic *Hfx. volcanii* exhibits a broad salt and temperature tolerance. The strain is able to grow at 0.7 M up to 5 M NaCl, with an optimal salt concentration of 2.2 M NaCl (Jantzer et al., 2011). The temperature range extends from 23 to 49°C (Robinson et al., 2005). To thrive in hypersaline habitats, *Hfx. volcanii* adjusts the intracellular salt concentration to the external salt concentration of the media (Christian and Waltho, 1962; Dennis and Shimmin, 1997; Oren, 1999). In general, the switching ability of riboswitch E was found to increase with rising salt concentrations. A threefold increase in *bgaH* expression was detected at 3 M NaCl, a fourfold increase at 3.5 M NaCl, and an even 12-fold increase was detected at 4 M NaCl in the presence of theophylline (Figure 4B). The expression was reduced to background expression without theophylline, and a proportional increase was observed with the rising salt concentrations in the presence of theophylline. Thus, at extremely high salt concentrations, the OFF and ON conformation of riboswitch E appears to be stabilized, resulting in an increased switching ability of the riboswitch (Supplementary Figure 6). Moreover, the switching ability of riboswitch E was also affected by temperature in *Hfx. volcanii* (Figure 4A), since an increase in the cultivation temperature resulted in the decrease of its switching ability. The highest activity was observed at 30°C with a 26-fold activation after supplementation of theophylline. Higher cultivation temperatures had a negative effect on the activation of riboswitch E in *Hfx. volcanii* although these temperatures are optimal for growth. One possible reason could be the misfolding of the riboswitch at higher temperatures.

Overall, a cultivation temperature of 30°C and 4 M NaCl turned out to be the best parameters for the application of theophylline-dependent riboswitch E in haloarchaea. Unfortunately, these are not the optimal conditions for the cultivation of *Hfx. volcanii* and result in a longer generation time. Therefore, a cultivation temperature of 37°C and a salt concentration of 3 M should be selected for the application of the theophylline-dependent riboswitch E in *Hfx. volcanii*. This makes the application of riboswitch E somewhat limited. It might be possible to optimize the switching activity of riboswitch E. Using bioinformatic modeling we tried to optimize the activity, but did not yet succeed (data not shown). The structure of the ON and OFF state of riboswitch E implies that this is not that easy (Supplementary Figure 6). The six different theophylline-dependent riboswitches used in this report are already optimized for use in different bacteria, but most of them were inactive in *Hfx. volcanii*. Riboswitch C was open in the presence or absence of theophylline, although the SD sequence is close to the AUG start and only 50% identical to the consensus sequence. Otherwise the sequence is identical. To achieve a better activity, multiple nucleotide alterations might be required.

### A Genetic Circuit Based on Riboswitch E and mGFP6 in Haloarchaea

Genetic circuits allow the controlled synthesis of specific proteins at a given stage of growth. This has the advantage to study the function of proteins at a desired growth stage in the cell. The amount of a regulatory protein plays a crucial role in the activation or repression of gene expression. To study the effect of increasing amounts of a regulator protein on expression, a dose-dependent gene expression system might be helpful. Riboswitch E was applied to study the GvpE-mediated activation of *P*_*pA*_ by regulating the *gvpE* expression in a theophylline-dependent manner. Plasmid pPAJB20 contained riboswitch E upstream of the *gvpE* reading frame, and in addition the *mgfp6* reporter under the control of promoter *P*_*pA*_ (Figure 5). The quantitation of the *P*_*pA*_ induction by GvpE in the presence or absence of theophylline yielded a low activity of *P*_*pA*_ in the absence of theophylline, and the mGFP6 fluorescence increased with rising theophylline concentrations up to 2 mM. The latter result underlined the data obtained using *bgaH* as reporter where an optimal induction was also reached at a concentration of 2 mM theophylline (Figure 3A). Western blot analysis confirmed an increase in the amount of GvpE with increasing theophylline concentrations. Moreover, similar amounts of GvpE were detected at theophylline concentrations of 2 and 3 mM. The expression derived from the riboswitch E-dependent GvpE-activation of *P*_*pA*_ was with 100.000 AU approximately half as high as the GvpE-activation of *P*_*pA*_ when the GvpE activator is produced from a leaderless transcript transcribed under *P*_*fdx*_ control. A reason for the lower expression is that the presence of a riboswitch always results in a leader-containing *gvpE* transcript, and leader-containing transcripts are less efficiently translated compared to leaderless transcripts (Sartorius-Neef and Pfeifer, 2004). In general, this experiment showed an activation of *P*_*pA*_ already at a low *gvpE* expression as well as a dependence of *P*_*pA*_ activation on the amount of GvpE. Earlier studies yielded similar results for mcGvpE derived from *Hfx. mediterranei*, where a high stimulation of the *P*_*mcA*_ promoter was observed despite of a drastic reduction of the amount of GvpE by GvpD (Zimmermann and Pfeifer, 2003).

## Conclusion

The data obtained in this report shows that the theophylline-dependent riboswitch E can be used in haloarchaea for the regulation of gene expression despite of the high intracellular salt concentrations. Integration of the mGFP6 reporter system with the theophylline-dependent *gvpE* expression allowed the analysis of the effects of different levels of *gvpE* expression on the induction of *P*_*pA*_. These new tools can be combined to study the gene expression in haloarchaea. Also, we demonstrated the regulatory potential of 5′UTRs and the potential of riboswitch E for conditional gene expression in haloarchaea. To exploit this potential, in addition to mutational analyses of the theophylline-dependent riboswitch E, other riboswitches, such as the neomycin- or ciprofloxacin-dependent riboswitch, should be investigated. Also, ribozymes or RNA thermometers (Narberhaus et al., 2006; Zhang et al., 2017) could be of interest for conditional gene expression in haloarchaea.

## Data Availability Statement

The original contributions presented in the study are included in the article/Supplementary Material, further inquiries can be directed to the corresponding author/s.

## Author Contributions

JB and FP planned the study. JB and KW performed the molecular biology analyses. FP, BS, and JB discussed the results and wrote the manuscript. All authors approved the final manuscript.

## Conflict of Interest

The authors declare that the research was conducted in the absence of any commercial or financial relationships that could be construed as a potential conflict of interest.
