# Supplementary material for: A Synthetic Riboswitch to Regulate Haloarchaeal Gene Expression
Source: Front Microbiol. 2021 Jun 15;12:696181. doi: 10.3389/fmicb.2021.696181 (PMC8241225; doi:10.3389/fmicb.2021.696181)
Supplement: Supplementary file 1 [file Data_Sheet_1.pdf]

Supplementary Material

A synthetic riboswitch to regulate haloarchaeal gene expression

Johannes Born[1], Kerstin Weitzel[1], Beatrix Suess[2,3], and Felicitas Pfeifer[1]

[1] Microbiology and Archaea, [2] Synthetic RNA Biology, Department of Biology, Technical University Darmstadt; [3] Centre of Synthetic Biology, Technical University Darmstadt, Darmstadt, Germany

*** Correspondence:**
Prof. Dr. Felicitas Pfeifer
pfeifer@bio.tu-darmstadt.de

Table S1. Plasmids used in this study

Plasmid	Description	Reference
pP$_{fdx}$JB18	Haloarchaeal expression vector containing *smRS-gfp* under control of P_{fdx}.	Born and Pfeifer, 2019
pPDPAJB18	Haloarchaeal expression vector containing *mgfp6* under control of P_{pA} (+ UAS$_A$)	Born and Pfeifer, 2019
pPDPAJB18+E	Haloarchaeal expression vector containing *mgfp6* under control of P_{pA} (+ UAS$_A$) and in the other direction *gvpE* under control of P_{fdx}.	Born and Pfeifer, 2019
pLacZJB20	Haloarchaeal expression vector containing upstream of *bgaH* the *lacZ* reading frame	This report
pFDXJB20_Eco	Haloarchaeal expression vector containing *bgaH* under control of P_{fdx}, *bgaH* (with ATG) and P_{fdx} are separated by an *Eco*RI site and a *Bam*HI site.	This report
pFDXJB20 _Eco_ΔATG	Haloarchaeal expression vector containing *bgaH* under control of P_{fdx}, *bgaH* and P_{fdx} are separated by an *Eco*RI site and a *Bam*HI site. Lacks the original ATG start of *bgaH* and was used to insert the riboswitch sequences containing the new ATG start.	This report
pFDXJB20_R	Haloarchaeal expression vector containing *bgaH* under control of P_{fdx} and one of the six theophylline riboswitches (R) controlling the expression.	This report
pFDXJB20 _CAA	Haloarchaeal expression vector containing *bgaH* under control of P_{fdx}. Contains CAA-repeats instead of the aptamer domain of (R).	This report
pFDXJB20	Haloarchaeal expression vector containing *bgaH* under control of P_{fdx}. P_{fdx} and *bgaH* are separated by a *Bam*HI site. Used to study the expression of leaderless *bgaH* transcripts.	This report
pPAJB20	Haloarchaeal expression vector containing *mgfp* under control of P_{pA} and in opposite direction *gvpE* under control of riboswitch E and P_{fdx}..	This report

Table S2. Oligonucleotides used in this study.

Name	Sequence (5' – 3')
Oligonucleotides for the generation of pLacZJB20	
pLacJB19_1_fwd	AAAAGGAATTCCACTGCAGAG
pLacJB19_1_rev	TCTGCAGTGGAATTCCTTTTGGATCCATGACAGTTGGTG
pLacJB19_2_fwd	CGTGACAGCCGAACTCGTTGTCAGTCTG
pLacJB19_2_rev	CAACGAGTTCGGCTGTCACGAGACGGTTAC
pLacJB19_3_fwd	GATGCTTCGCGGTATCCGCGGTCCGGAG
pLacJB19_3_rev	CGCGGATACCGCGAAGCATCGACCGCCG
pLacJB19_4_fwd	GTTCCTCCTTTCAGATGGGTACCGGTACCTCACTCGGACGCG
pLacJB19_4_rev	GGTACCCATCTGAAAGGAGGAACTATATCC
Oligonucleotides for insertion of P_{fdx} sequence including the *Eco*RI recognition site in pLacZJB20 (resulting in pFDXJB20_Eco). The *Nco*I and *Bam*HI overhangs are in bold.	
FDX+EcoRI_fwd	**CATGG**CGGGCTTTCGTGGCAGTACGCTGGCCCGAACAGCAACTACTATGC<br>GTTCGGAAGCCGAACTCTGCAGTGAATTCTTAATA**G**
FDX+EcoRI_rev	**GATCC**TATTAAGAATTCACTGCAGAGTTCGGCTTCCGAACGCATAGTAGT<br>TGCTGTTCGGGCCAGCGTACTGCCACGAAAGCCCG**C**
Oligonucleotides for deletion of the ATG start of the *bgaH* reading frame in pFDXJB20_Eco (resulting in pFDXJB20_Eco_ΔATG)	
ΔATG-bagH_fwd	ACAGTTGGTGTCTGCTATTTCCCGGAGCACT
ΔATG-bagH_rev	GGATCCTTATTTTGACACCAGACCAACTGGTAATG
Oligonucleotides for insertion of P_{fdx} in pLacZJB20 (resulting in pFDXJB20). The *Nco*I and *Bam*HI overhangs are in bold.	
FDX_fwd	**CATGG**CGGGCTTTCGTGGCAGTACGCTGGCCCGAACAGCAACTACTATGC<br>GTTCGGAAGCCGAACTCTGCAGT**G**
FDXI_rev	**GATCC**ACTGCAGAGTTCGGCTTCCGAACGCATAGTAGT<br>TGCTGTTCGGGCCAGCGTACTGCCACGAAAGCCCG**C**
Oligonucleotides for insertion of the constant region and respective riboswitches in pLacZJB20_Eco_ΔATG. The *Eco*RI and *Bam*HI overhangs are in bold.	
RA_fwd	**AATTC**ATACGACTCACTATAGGTTCGGTGATACCAGCATCGTCTTGATGCC<br>CTTGGCAGCACCTGAGAAGGGGCAACAAGATG**G**
RA_rev	**GATCC**CATCTTGTTGCCCCTTCTCAGGTGCTGCCAAGGGCATCAAGACGAT<br>GCTGGTATCACCGAACCTATAGTGAGTCGTAT**C**
RB_fwd	**AATTC**ATACGACTCACTATAGGTTCCGGTGATACCAGCATCGTCTTGATGC<br>CCTTGGCAGCACCCGCTGCGCAGGGGGTATCAACAAGATG**G**
RB_rev	**GATCC**CATACGACTCACTATAGGTTCGGTGATACCAGCATCGTCTTGATGC<br>CCTTGGCAGCACCTGAGAAGGGGCAACAAGATGGGATC**C**

RC_fwd	**AATTC**ATACGACTCACTATAGGTTCCTGATAAGATAGGGGTGATACCAGC ATCGTCTTGATGCCCTTGGCAGCACCAAGGGACAACAAGATG**G**
RC_rev	**GATCC**CATCTTGTTGTCCCTTGGTGCTGCCAAGGGCATCAAGACGATGCTG GTATCACCCCTATCTTATCAGGAACCTATAGTGAGTCGTAT**C**
RD_fwd	**AATTC**ATACGACTCACTATAGGTTCCGGTGATACCAGCATCGTCTTGATGC CCTTGGCAGCACCCTGCTAAGGTAACAACAAGATG**G**
RD_rev	**GATCC**CATCTTGTTGTTACCTTAGCAGGGTGCTGCCAAGGGCATCAAGAC GATGCTGGTATCACCGGAACCTATAGTGAGTCGTAT**C**
RE_fwd	**AATTC**ATACGACTCACTATAGGTTCCGGTGATACCAGCATCGTCTTGATGC CCTTGGCAGCACCCTGCTAAGGAGGTAACAACAAGATG**G**
RE_rev	**GATCC**CATCTTGTTGTTACCTCCTTAGCAGGGTGCTGCCAAGGGCATCAAG ACGATGCTGGTATCACCGGAACCTATAGTGAGTCGTAT**C**
RE*_fwd	**CATGG**ATACGACTCACTATAGGTTCCGGTGATACCAGCATCGTCTTGATG CCCTTGGCAGCACCCTGCTAAGGAGGCAACAAGATG**G**
RE*_rev	**GATCC**CATCTTGTTGCCTCCTTAGCAGGGTGCTGCCAAGGGCATCAAGAC GATGCTGGTATCACCGGAACCTATAGTGAGTCGTAT**C**
CAA_fwd	**CATGG**ATACGACTCACTATAGGTTCCCAACAACAACAACAACAACAACAA CAACAACAACAACACTGCTAAGGAGGCAACAAGATG**G**
CAA_rev	**GATCC**CATCTTGTTGCCTCCTTAGCAGTGTTGTTGTTGTTGTTGTTGTT GTTGTTGTTGTTGGGAACCTATAGTGAGTCGTAT**C**

Oligonucleotides for deletion of the ATG start of c-*gvpE* in pPAJB20

ΔATG-E_fwd	GACGACCTCTTAGCGGAGCTCAA
ΔATG-E_rev	ACTAGTCTGCAGAGTTCGGCTTCC

Oligonucleotides for insertion of the *Eco*RI recognition site in pPAJB20 (*Eco*RI site in bold). The spacer between the recognition sequences of *Eco*RI and *Spe*I is underlined.

EcoRI_fwd	**GAATTC**TATATA**ACTAGT**GACGACCTCTTAGCGG
EcoRI_rev	ACTAGTCTGCAGAGTTCGGCTTCC

Oligonucleotides to insert riboswitch E in pPAJB20. The *Eco*RI and *Spe*I overhangs are in bold.

RE_fwd	**AATTC**ATACGACTCACTATAGGTTCCGGTGATACCAGCATCGTCTTGATGC CCTTGGCAGCACCCTGCTAAGGAGGTAACAACAAGATG**A**
RE_rev	**GATCA**CATCTTGTTGTTACCTCCTTAGCAGGGTGCTGCCAAGGGCATCAAG ACGATGCTGGTATCACCGGAACCTATAGTGAGTCGTAT**C**

Table S3. Switching activity of the different riboswitch sequences in *Hfx. volcanii*.

Transformant	basal activity*	+ theophylline	activation factor
	Miller Units [MU]		
FDX	15.40 ± 1.02	16.00 ± 1.20	/
CAA	4.62 ± 0.16	4.40 ± 0.14	1
RA	0.01 ± 0.00	0.01 ± 0.00	1
RB	0.02 ± 0.00	0.02 ± 0.00	1
RC	0.95 ± 0.03	0.93 ± 0.05	1
RD	0.05 ± 0.00	0.05 ± 0.00	1
RE	0.10 ± 0.02	0.31 ± 0.06	3
RE*	0.02 ± 0.00	0.02 ± 0.01	1

*The experiments were performed in three biological and three technical replicates. Growth was at 37°C in 3 M NaCl medium in the absence (basal activity) or in the presence of 2 mM theophylline.

Table S4. Influence of salt and temperature on the activation of riboswitch E in *Hfx. volcanii.*

Condition	basal activity*	+ theophylline	activation factor (x-fold)
	Miller Units [MU]		
Temperature [°C], 3 M NaCl			
30	0.06 ± 0.00	1.53 ± 0.15	26
37	0.19 ± 0.02	0.57 ± 0.05	3
42	0.16 ± 0.04	0.25 ± 0.01	1.5
45	0.10 ± 0.03	0.12 ± 0.03	1
Salt concentration, 37°C			
1.5 M	0	0	-
2.0 M	0	0	-
3.0 M	0.17 ± 0.01	0.54 ± 0.11	3
3.5 M	0.24 ± 0.03	1.02 ± 0.13	4
4.0 M	0.09 ± 0.00	1.15 ± 0.09	12
30°C, 4 M NaCl	0.30 ± 0.01	4.00 ± 0.48	13

*The experiments were performed in three biological and three technical replicates, in the absence (basal activity) or in the presence of 2 mM theophylline.

Table S5. Activation of P_A depending on different amounts of GvpE in PAJB20 transformants.

Theophylline concentration	GvpE-induced Fluorescence [AU] x 10^3	activation factor (x-fold)
0 mM	30 ± 3	0
1 mM	70 ± 4	2.3
2 mM	105 ± 6	3.5
3 mM	101 ± 7	3.3

*The experiments were performed in three biological and three technical replicates at 37°C in 3 M NaCl medium.

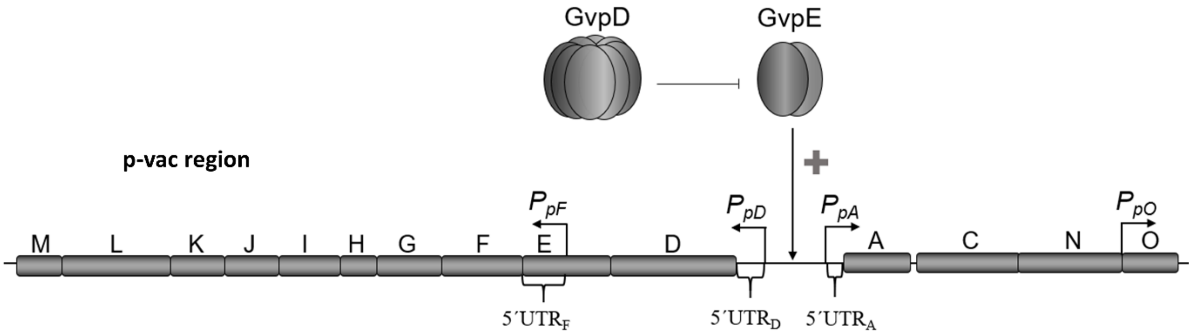

Figure S1. Regulation of transcription of the p-vac region. The 14 *gvp* genes are shown as boxes labelled A and C through O. The transcription is driven by the four promoters P_{pA}, P_{pD}, P_{pF} and P_pO, and the direction of transcription is indicated by arrows. The activities of P_{pA} and P_{pD} are enhanced by the endogenous activator GvpE. GvpD and GvpE interact, leading to a decrease in the amount of GvpE, resulting in a lower expression. The p-*gvpACNO*, p-*gvpDE*, and p-*gvpFGHIJKLM* transcripts contain 5′UTRs of 20, 72, and 169 nt, respectively. The p-*gvpO* transcript is leaderless.

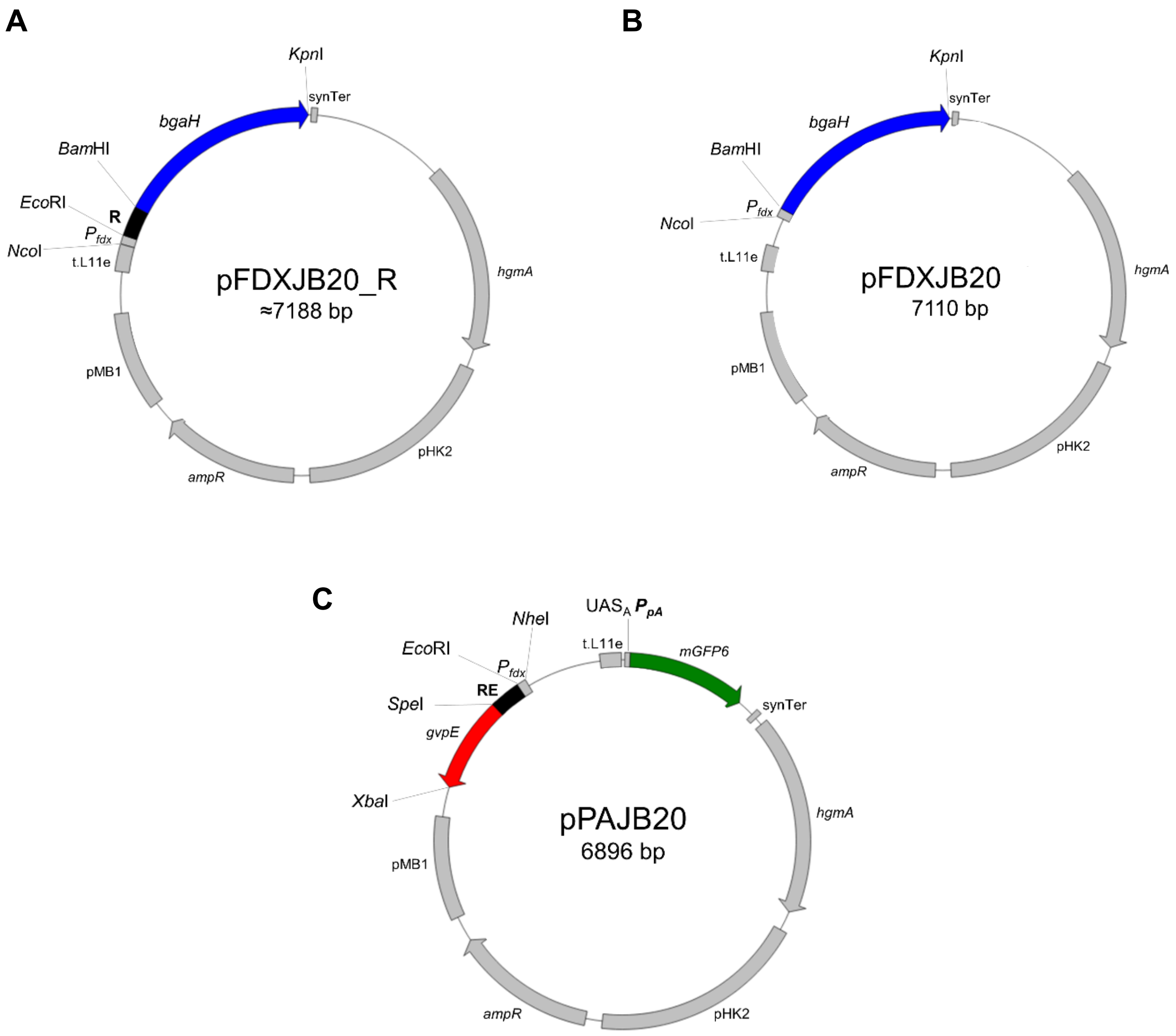

Figure S2. Shuttle vectors constructed in this study. The *Escherichia coli* portion consists of plasmid pMB1 and ampR (β-lactamase for selection by ampicillin), and the *Haloferax volcanii* portion of plasmid pHK2 and *hmgA* (HMG-CoA reductase for selection by mevinolin or lovastatin). The genes of interest are colored. **(A)** Expression vector pFDXJB20_R. The plasmid was used to test riboswitch A through E and E* in *Hfx. volcanii*. One of the six riboswitches, or the CAA repeat, was placed between the *Eco*RI and *Bam*HI sites upstream of *bgaH* (R, black). The transcription of *bgaH* (blue) is driven by the strong P_{fdx} promoter. The reporter gene is flanked by the archaeal terminator t.L11e and the synthetic terminator synTer. **(B)** Expression vector pFDXJB20 contains P_{fdx} directly upstream of the *bgaH* reading frame to generate leaderless transcripts. **(C)** Expression vector pPAJB20. The vector contains a small genetic circuit to investigate *mgfp6*-expression depending on external concentrations of theophylline. The expression of *gvpE* (red) is under control of the theophylline-dependent riboswitch E (RE) and P_{fdx}. The reporter gene *mgfp6* (green) is placed in opposite direction under control of the P_{pA} promoter including the UAS_A sequence required for GvpE activation. The *gvpE* gene and *mgfp6* are separated by the terminator t.L11e.

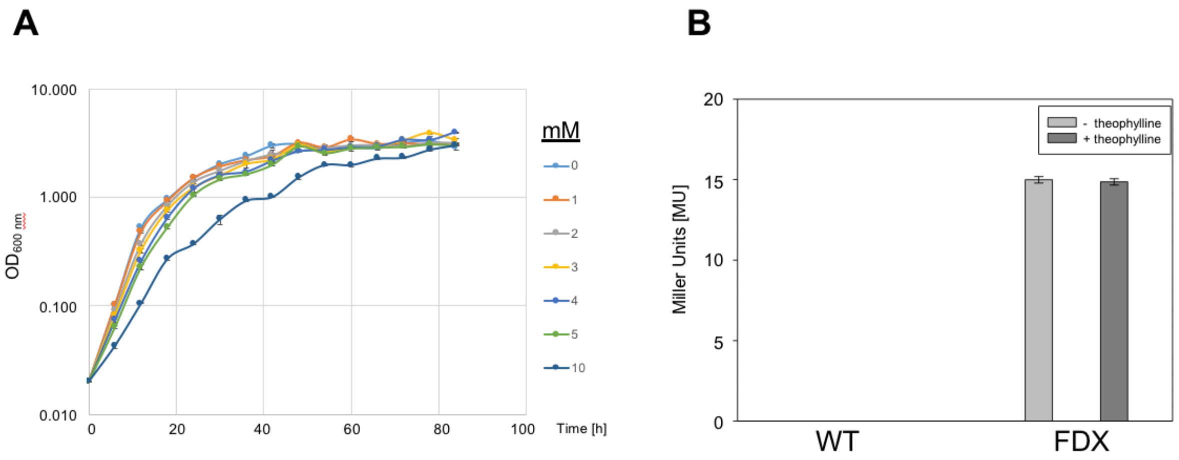

Figure S3. Effect of theophylline on *Hfx. volcanii* and expression studies. (**A**) Influence of different theophylline concentrations on the growth of *Hfx. volcanii* WR340 wild type. Cultures were incubated for 84 h in 3 M salt media containing 1, 2, 3, 4, 5, or 10 mM theophylline at 42°C. The optical density was measured at different time points and the values were plotted semi-logarithmically. The experiments were performed in triplicates on three different days. (**B**) Effect of theophylline on *bgaH* expression in *Hfx. volcanii* pFDXJB20 transformants. The wild type (WT) lacking the plasmid containing *bgaH* served as negative control. Plasmid pFDXJB20 contains the *bgaH* reading frame under control of P_{fdx}. Cells were grown at 42°C in media containing 3 M NaCl in the presence of absence of 2 mM theophylline. The BgaH activity was determined after 36 h (OD_{600} 1.2) and expressed in Miller units. Both experiments were performed in triplicates on three different days.

A

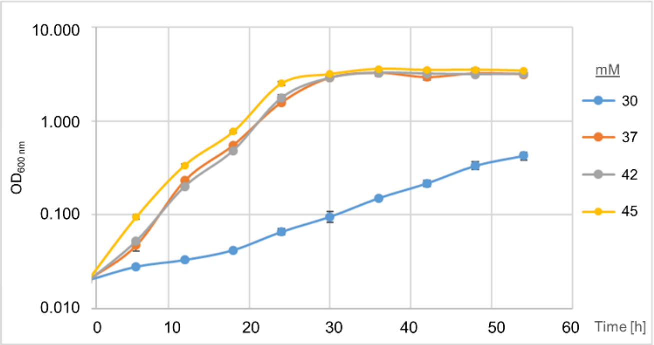

B

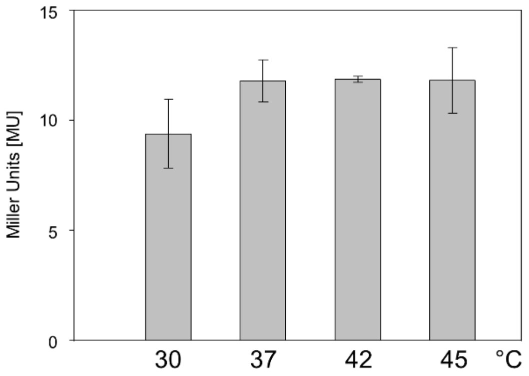

Figure S4. Effect of temperature on growth and on the *bgaH* expression in *Hfx. volcanii*. (**A**) Effect of cultivation temperature on the growth of *Hfx. volcanii* wild type. Cultures were incubated for 54 h at 30, 37, 42, or 45°C in 3 M salt media, and the optical densities were measured at different time points. The values were plotted semi-logarithmically. (**B**) Effect of temperature on the expression in *Hfx. volcanii* pFDXJB20 transformants. The transformants were grown at 30, 37, 42, or 45°C in 3 M salt media, and after 45 h of growth samples were taken and the BgaH activity determined. Both experiments were performed in triplicates on three different days.

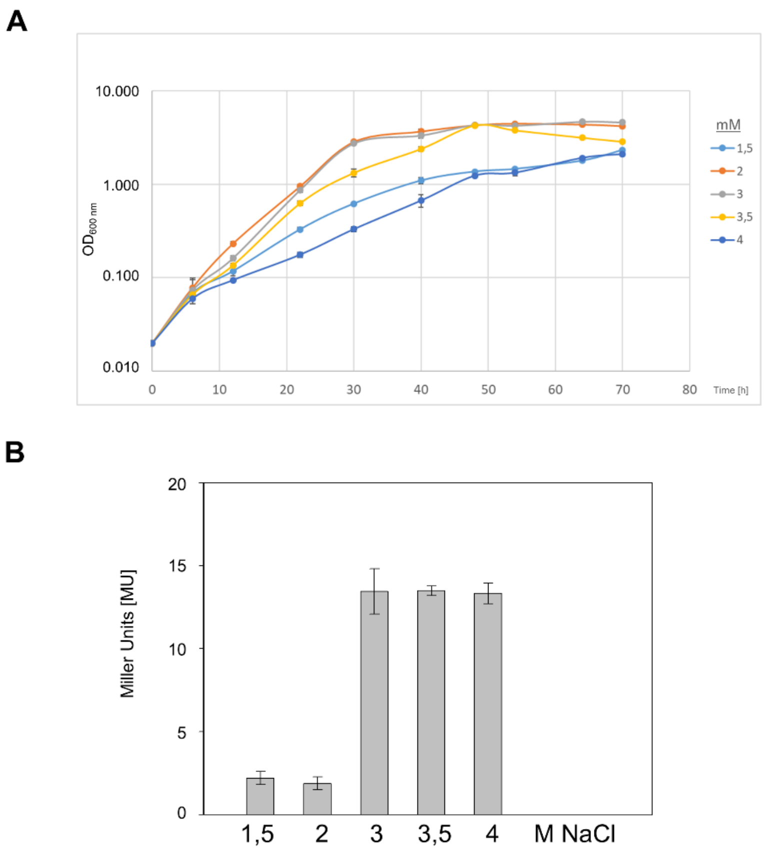

Figure S5. Influence of the salt concentration on growth and *bgaH* expression in *Hfx. volcanii*. (**A**) Effect of salt concentration on the growth of *Hfx. volcanii* wild type. Cultures were incubated for 70 h in 1.5; 2.0; 3.0; 3.5, or 4.0 M NaCl containing media at 37°C, and the optical densities were determined at different time points. The values were plotted logarithmically. (**B**) Effect of salt concentration on the expression of *bgaH* in *Hfx. volcanii* transformants. Transformants containing pFDXJB20 were cultured in 1.5; 2.0; 3.0; 3.5, or 4.0 M salt media at 37°C. After 37 h of growth the BgaH activity was determined in Miller units. Both experiments were performed in triplicates on three different days.

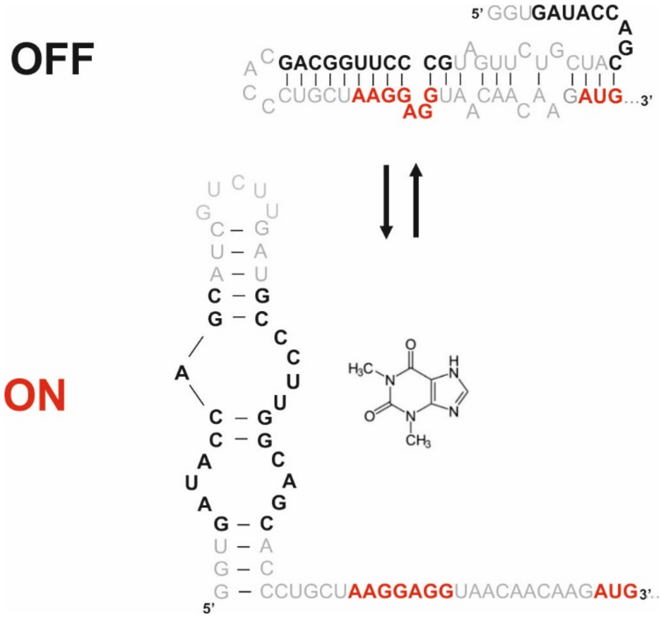

Figure S6. Sequence and secondary structure of the theophylline-dependent riboswitch E. The OFF and ON state of the riboswitch is shown. The AUG start codon and the Shine-Dalgarno sequence are shown in red. The nucleotides interacting with theophylline are shown in black and bold. Binding of theophylline induces the ON state and the access of SD sequence and AUG start codon.
